# Identification of a novel canine parvovirus type 2c in Taiwan

**DOI:** 10.1186/s12985-016-0620-5

**Published:** 2016-09-23

**Authors:** Shu-Yun Chiang, Hung-Yi Wu, Ming-Tang Chiou, Min-Chen Chang, Chao-Nan Lin

**Affiliations:** 1 Department of Veterinary Medicine, College of Veterinary Medicine, National Pingtung University of Science and Technology, Pingtung, Taiwan; 2 Graduate Institute of Veterinary Pathobiology, College of Veterinary Medicine, National Chung-Hsing University, Taichung, Taiwan; 3 Animal Disease Diagnostic Center, College of Veterinary Medicine, National Pingtung University of Science and Technology, Pingtung, Taiwan

**Keywords:** Canine parvovirus, CPV-2c, Taiwan

## Abstract

**Background:**

Taiwan has been considered free from canine parvovirus type 2c (CPV-2c) based on the last report of canine parvovirus type 2 (CPV-2) surveillance. However, since January 2015, the first report of CPV-2c in a puppy has occurred in Taiwan. There is currently limited information about the CPV-2c variant in Taiwan. In the present study, we characterized the previously unidentified CPV-2c variant and investigated the distribution of CPV-2 variants in Taiwan.

**Methods:**

During January 2014 to April 2016, fecal or rectal swab samples from 99 dogs with suspected CPV-2 infection in Taiwan were collected. Eighty-eight were identified as being either CPV-2a, −2b or -2c variants positive by real-time PCR and sequence analysis.

**Results:**

Sequence analysis of the 88 isolates confirmed CPV-2c as the dominant variant (54.6 %), followed by CPV-2b (26.1 %) and CPV-2a (19.3 %). Phylogenetic analysis demonstrated that the recent CPV-2c variants are similar to the Chinese CPV-2c strain but can be considered as novel Asian CPV-2c isolates.

**Conclusion:**

The present study provides evidence for the existence of a novel CPV-2c variant in Taiwan.

**Electronic supplementary material:**

The online version of this article (doi:10.1186/s12985-016-0620-5) contains supplementary material, which is available to authorized users.

## Background

Canine parvovirus type 2c (CPV-2c) was first detected in Italy in 2000 [1]. Antigenic differences among CPV-2a, −2b, and -2c are observed only in residue 426 (Asn in 2a, Asp in 2b, and Glu in 2c) [2], which is located in the major VP2 antigenic site of the parvovirus [3]. The functions of capsid protein VP2 include facilitating receptor binding, controlling host range [4], and eliciting neutralizing antibodies [3]. Although CPV-2c infection results in almost the same clinical signs as for CPV-2a and CPV-2b, including anorexia, vomiting, acute gastroenteritis, and hemorrhagic diarrhea, infection by CPV-2c has been reported to be indicative of a more severe disease [5, 6].

A retrospective analysis revealed that the oldest CPV-2c strain was identified in Germany in 1996 [7]. Another retrospective analysis revealed that the frequency of CPV-2 variants underwent rapid fluctuation in Italy between 1995 and 2005, with CPV-2c very rapidly replacing CPV-2b [8]. CPV-2c infection has not only been observed in Italy, but it is also widely distributed in other European countries [7, 9, 10], including Germany, Portugal [11], Spain [12], Belgium, France, Greece [13], Bulgaria [14], Sweden [15], Turkey [16], and the United Kingdom. In recent years, CPV-2c has also been found to be widespread in Tunisia [17], the USA [18], Uruguay [19], Brazil [20], Argentina [21], Ecuador [5], Mexico [22], and Morocco [23]. Surprisingly, since the first reported finding in Vietnam in 2004, the CPV-2c variant has not been prevalent in Asia [24]. Indeed, only a few CPV-2c strains have been isolated in India [25] and China [26–28], with either CPV-2a or -2b being prevalent in Asian countries thus far [25–27, 29–42].

In Taiwan, as in other Asian countries, both the CPV-2a and -2b genotypes constitute the prevalent CPV-2 field strains circulating in the last two decades [30, 31, 39, 41]. Before the present study, no report indicated the occurrence of a CPV-2c variant in Taiwan [30, 31, 39, 41]. However, in January 2015, the first report of CPV-2c in a puppy in Taiwan occurred, and there is to date limited information about the CPV-2c variant in Taiwan. In the present study, we examined this CPV-2c variant and investigated the distribution of CPV-2 variants in Taiwan.

## Methods

### Specimen collection

Clinical specimens (feces and/or rectal swab) were collected from 99 dogs with suspected CPV-2 infection from northern, central, southern, and eastern Taiwan from January 2014 to April 2016. These samples were mainly acquired from dogs with diarrhea and/or bloody diarrhea. The year of sampling and the age, clinical history, and CPV-2 types of the sampled dogs are summarized in Additional file 1.

### CPV-2 screening and partial VP2 gene amplification

Viral DNA was extracted from the clinical samples (either feces or rectal swab) and screened for CPV-2 by real-time PCR, as described by Lin et al. [43]. Samples showing positive results for either type of specimen were included in this study. The partial VP2 gene of CPV-2 was amplified by PCR, as described by Buonavoglia et al. [1], and the DNA fragments were purified and sequenced as described by Lin et al. [41].

### Sequence and phylogenetic analyses

The VP2 DNA sequences of our samples were compared to those of reference FPV (M38246), CPV-2 (M38245), CPV-2a (M24003), CPV-2b (M74849), new CPV-2a (JX048605), new CPV-2b (JX048607), and CPV-2c (JF414818, JF414820, JF414822, FJ005247, GU380303, GU380305, KR611522, KT074339, KT162005, KF149962, FJ005196, GQ865518, FJ222821, FJ005213, FJ005238, FJ005214, KC196099, KM457119, AB120727, KP071956, KR559893, FJ005235). Multiple alignments of the nucleic acid and amino acid sequences were performed using the Clustal W method and the MegAlign program (DNASTAR, Madison, WI, USA). Phylogenetic analyses were performed with the maximum likelihood method using MEGA 6, version 6.06.

## Results

### Polymerase chain reaction (PCR) amplification and genotype analysis

**Fig. 1:**
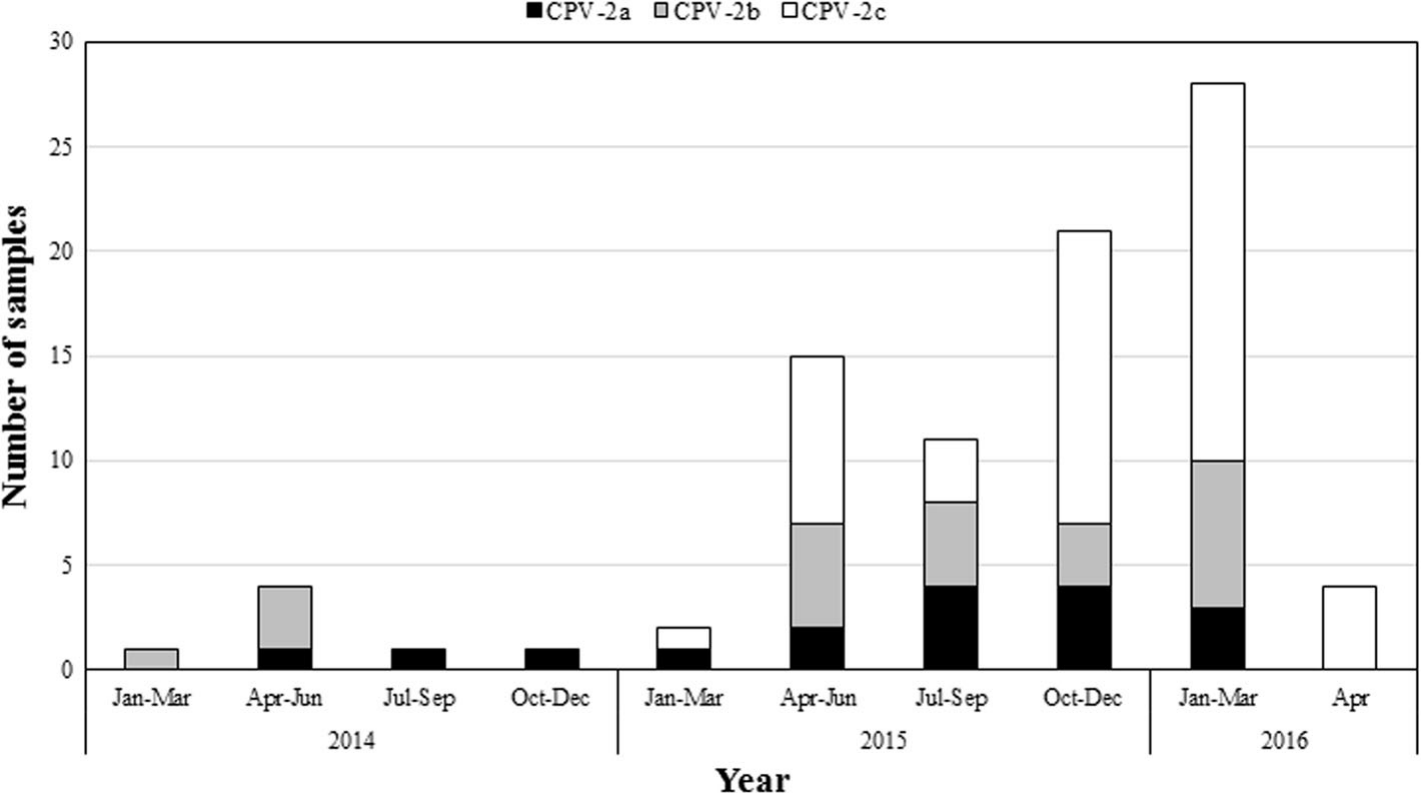
Distribution of CPV-2 variants in Taiwan according to month between January 2014 and April 2016

A total of 88 samples from 99 dogs were positive for CPV-2. Of the 88 CPV-2 isolates, 17, 23, and 48 were identified as CPV-2a (19.3 %), CPV-2b (26.1 %), and CPV-2c (54.6 %), respectively. First, we observed the CPV-2c variant collected in January 2015 (Additional file 1, Fig. 1). This variant rapidly spread throughout the Taiwanese dog population, and detection rates were 53.1 % (26/49) and 68.8 % (22/32) in 2015 (January to December) and 2016 (January to April), respectively (Fig. 1). Taken together, our data revealed co-circulation of CPV-2a, −2b, and -2c on the island, and CPV-2c was identified as the current dominant variant.

### Geographical distribution of the CPV-2 variants

**Fig. 2:**
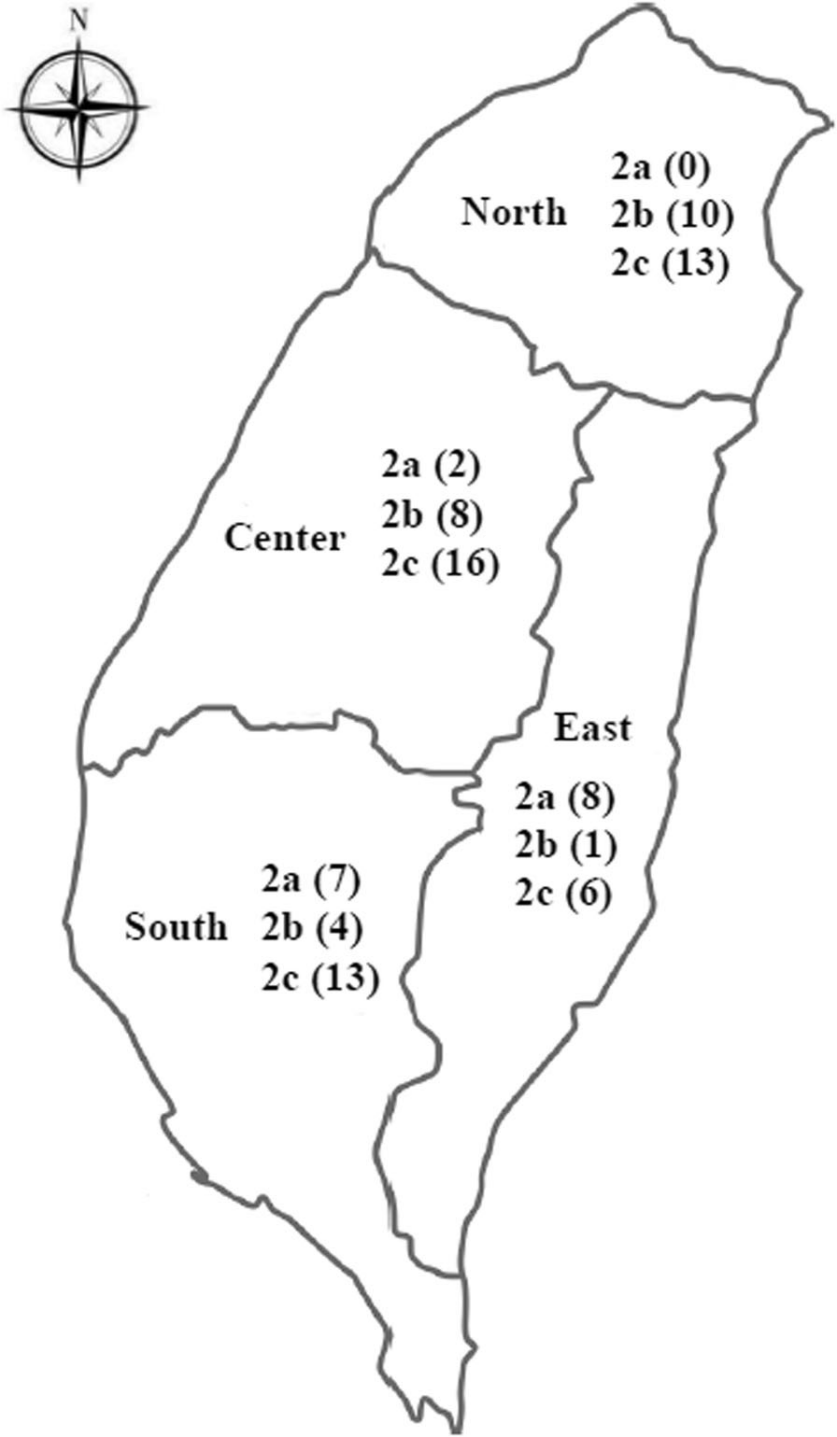
Geographical distribution of CPV-2 variants collected from the dog population between January 2014 and April 2016

Of the 17 CPV-2a variants, 2 (11.8 %), 7 (41.2 %), and 8 (47.0 %) isolates were collected from central, southern, and eastern Taiwan, respectively (Fig. 2). Of the 23 CPV-2b variants, 10 (43.5 %), 8 (34.8 %), 4 (17.4 %), and 1 (4.3 %) isolates were collected from northern, central, southern, and eastern Taiwan, respectively (Fig. 2). Of the 48 CPV-2c variants, 13 (27.1 %), 16 (33.3 %), 13 (27.1 %) and 6 (12.5 %) isolates were collected from northern, central, southern, and eastern Taiwan, respectively (Fig. 2). Taken together, our results showed distribution of the CPV-2c variant throughout Taiwan.

### DNA sequence analysis

Partial VP2 nucleotide sequences were analyzed using DNASTAR software, revealing 96.6–100 %, 98.3–100 %, and 98.5–100 % homology within local CPV-2a isolates, CPV-2b isolates, and CPV-2c isolates, respectively (Table 1). The same nucleotide sequences for local CPV-2c isolates exhibited 96.9–99 % and 97.5–99.2 % homology with those of the CPV-2a and -2b isolates, respectively (Table 1). In contrast to the low nucleotide sequence similarity between prototype CPV-2c (FJ222821, from Italy) and CPV-2c from Taiwan (97.9–99.0 %), the homology levels between our analyzed CPV-2c isolates (97.7–100 %) and Chinese CPV-2c (GU380303, GU380305, KR611522, KT074339, KT162005) appeared to be much higher (Table 1).

**Table 1:** Sequence homology of Taiwanese CPV-2 variants and reference strains

Strains	Genotypes		
	Taiwanese CPV-2a	Taiwanese CPV-2b	Taiwanese CPV-2c
Taiwanese CPV-2a	96.6–100.0	98.3–99.6	96.9–99.0
Taiwanese CPV-2b		98.3–100.0	97.5–99.2
Taiwanese CPV-2c			98.5–100.0
Chinese CPV-2c^a^			97.7–100.0
Italy CPV-2c (FJ222821)			97.9–99.0

^a^Accession numbers: GU380303, GU380305, KR611522, KT074339, KT162005

### Amino acid sequence analysis

Comparisons among the 88 isolates and 12 reference strains at amino acids 267–440 are presented in Table 2. For the partial VP2 analysis, 17 sequences with an Asn at position 426 were classified as CPV-2a. All of the CPV-2a strains show substitution at position 324 (Tyr to Ile) caused by mutation of TAT to ATT at nucleotide positions 970–972 of the VP2 gene (Table 2). Interestingly, five CPV-2a strains from eastern Taiwan are identical in nucleotide sequence but have distinctive residues (Phe267Tyr, Tyr324Ile, and Thr440Ala) (Table 2). Four CPV-2b strains are identical to the prototype of CPV-2b (Table 2). Fifteen of the 19 CPV-2b strains show substitution at position 267 (Phe to Tyr) and 324 (Tyr to Ile), and four CPV-2b strains only show substitution at position 324 (Tyr to Ile). Glu426, which is unique to strain CPV-2c, was first observed in 48 samples in this study. One unique amino acid substitution was found in the all CPV-2c isolates (Gln370Arg) (Table 2), which is caused by mutation of CCA to CGA at nucleotide positions 1,108-1,110 of the VP2 gene. All CPV-2c variants also show amino acid substitution at position 267 (Phe to Tyr) and 324 (Tyr to Ile). Interestingly, 10 of 48 CPV-2c variants show a unique substitution on Phe420Ser.

**Table 2:** Amino acid mutations of the VP2 gene sequences analyzed in this study

Isolate	Amino acid at position						
	267	297	324	370	420	426	440
CPV2 (M38245)	Phe	Ser	Tyr	Gln	Phe	Asn	Thr
Partial-length VP2							
CPV-2a-1^a^ (*n* = 12)	Phe	Ala	Ile	Gln	Phe	Asn	Thr
CPV-2a-2^b^ (*n* = 5)	Tyr	Ala	Ile	Gln	Phe	Asn	Ala
CPV-2b-1^c^ (*n* = 4)	Phe	Ala	Tyr	Gln	Phe	Asp	Thr
CPV-2b-2^d^ (*n* = 15)	Tyr	Ala	Ile	Gln	Phe	Asp	Thr
CPV-2b-3^e^ (*n* = 4)	Phe	Ala	Ile	Gln	Phe	Asp	Thr
CPV-2c-1^f^ (*n* = 38)	Tyr	Ala	Ile	Arg	Phe	Glu	Thr
CPV-2c-2^g^ (*n* = 10)	Tyr	Ala	Ile	Arg	Ser	Glu	Thr

^a^KX396349, KX396353, KX396354, KX396356, KX396359, KX396365, KX396375, KX396377, KX396378, KX396385, KX396388, KX396419

^b^KX396376, KX396390, KX396400, KX396404, KX396415

^c^KX396348, KX396361, KX396383, KX396420

^d^KX396368, KX396369, KX396370, KX396371, KX396372, KX396373, KX396374, KX396386, KX396387, KX396405, KX396406, KX396407, KX396409, KX396417, KX396421

^e^KX396350, KX396351, KX396352, KX396382

^f^KX396355, KX396357, KX396358, KX396360, KX396362, KX396363, KX396364, KX396366, KX396367, KX396379, KX396380, KX396381, KX396384, KX396389, KX396391, KX396392, KX396393, KX396394, KX396395, KX396396, KX396397, KX396399, KX396401, KX396402, KX396403, KX396408, KX396410, KX396411, KX396412, KX396413, KX396414, KX396422, KX396424, KX396425, KX396427, KX396433, KX396434, KX396435

^g^KX396398, KX396416, KX396418, KX396423, KX396426, KX396428, KX396429, KX396430, KX396431, KX396432

### Phylogenetic analysis

**Fig. 3:**
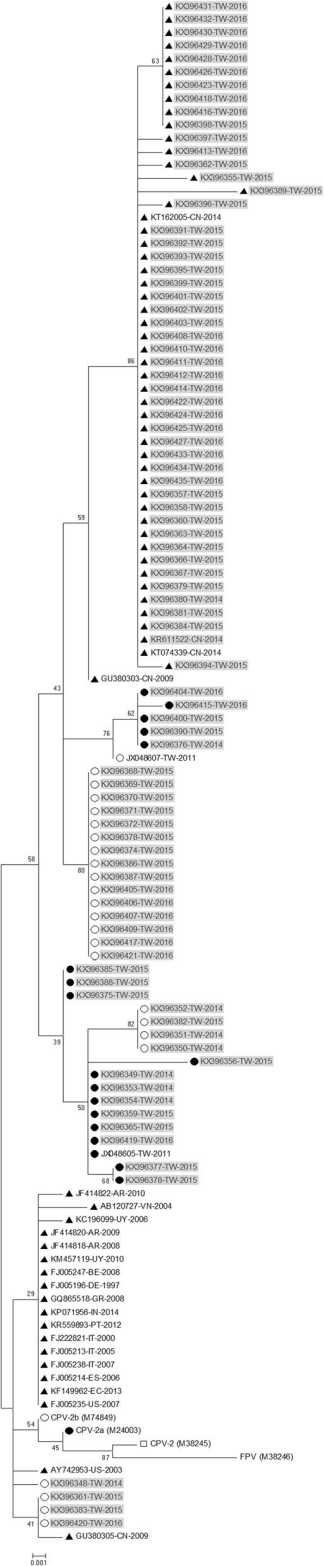
Phylogenetic analysis of nucleotide sequences of the partial VP2 genes of CPV-2 strains (522 bp). The phylogenetic tree was constructed using the maximum likelihood method with bootstrap analysis (*n* = 1,000) to determine the best fitting tree. CPV variants are indicated by □, ●, ○, and ▲ for CPV-2, 2a, 2b, and 2c, respectively. The gray diagram represents the Taiwanese CPV-2 variants in this study

The phylogenetic tree of the partial VP2 gene for the 88 isolates and 28 reference strains was generated using the maximum likelihood method with MEGA 6, version 6.06. Two clusters based on the phylogenetic relationship of the partial VP2 gene of CPV-2c were detected (Fig. 3). The first cluster comprises prototype isolates, European isolates, American isolates, and Asian isolates of CPV-2c. The second cluster consists of all 48 Taiwanese isolates from this study and 4 Chinese isolates of CPV-2c (Fig. 3). To date, these novel CPV-2c isolates have only been detected in China and Taiwan. Our findings demonstrated that the recent CPV-2c isolates in Taiwan are more genetically similar within the VP2 gene to Chinese CPV-2c strains than to prototype strains of CPV-2c.

## Discussion

This is the first study to demonstrate the CPV-2c variant in Taiwan. Previous studies have shown that both the CPV-2a and -2b genotypes constitute the prevalent CPV-2 field strains circulating in Taiwan, and no CPV-2c cases were reported in the last two decades (Table 3). In our continuous surveillance and sequence analysis, Taiwan was considered to be free of CPV-2c. However, since January 2015, one case of CPV-2c occurred in a puppy in Taiwan. The CPV-2c variant appears thus far to have the highest detection rate in the dog population of Taiwan. Similar results have shown that CPV-2c replaced the previous circulation of CPV-2 strains in Italy [8], Uruguay [19], Argentina [21], Brazil [20], and the United States [44]. Interestingly, comparative VP2 genome analysis of the isolated CPV-2c reference revealed that the partial VP2 genome sequence of the Taiwanese strain is similar (97.7–100 %) to that of Chinese CPV-2c strains. Our results indicate that the recent CPV-2c isolate from Taiwan shares a common evolutionary origin with the Chinese CPV-2c strains and should be classified as novel Asian CPV-2c isolates. According to genotype surveillance between Taiwan [30, 31, 39, 41] and China [26–28, 37, 45–48], CPV-2c was first detected in China in 2009 [28]. In contrast, no CPV-2c variant was observed in studies conducted over the last two decades in Taiwan (Table 3). In addition, the Taiwanese CPV-2c variant is more closely related to the Chinese CPV-2c strains than the recent Taiwanese CPV-2a (JX048605) and -2b (JX048607) isolates (Fig. 3). Taken together, our results suggest that the Taiwanese CPV-2c may be present due to import from China at some time between the end of 2014 to early 2015 rather than to evolution of existing CPV-2a or -2b genotypes. An ongoing investigation and complete VP2 genome sequence analysis is needed to trace the genetic evolution of this novel CPV-2c variant.

**Table 3:** Review of CPV-2 genotyping in Taiwan

Study period	Region of Taiwan	Genotype of CPV-2			References
		2a	2b	2c	
1994–1995	North	10	1	0	Chang et al., 1996 [30]
2003–2004	Central	2	34	0	Wang et al., 2005 [31]
2011	South	35	19	0	Chou et al., 2013 [39]
2008–2012	North, Central, and South	15	13	0	Lin et al., 2014 [41]
2014–2016	North, Central, South, and East	17	23	48	This study

The recent Taiwanese CPV-2a is composed of two divergent lineages that have different ancestors. Most Taiwanese CPV-2a strains belong to the recent Taiwanese lineage of CPV-2a, sharing a common amino acid substitution (Tyr324Ile). The second lineage of the Taiwanese CPV-2a variant is more closely related to recent Uruguayan and Chinese CPV-2a strains and has distinctive amino acid substitutions of Phe267Tyr, Tyr324Ile, and Thr440Ala. This new CPV-2a variant was discovered in China and Uruguay between 2006 and 2009 and in 2010 [49], respectively. This is the first detection of this lineage of CPV-2a in eastern Taiwan in 2015. However, this new CPV-2a variant recently emerged in Uruguay and underwent clonal expansion [49]. An ongoing investigation is aimed at determining whether this new CPV-2a variant will replaced the CPV-2c variant in the Taiwanese dog population.

Amino acid substitution of Tyr324Ile has been observed in Korea [32, 34], China [26, 27, 37, 45–48], Thailand [36], Uruguay [49, 50], Japan [38], Taiwan [39, 41], and India [40, 42]. Interestingly, the frequency of the Ile324 variant has reached a high prevalence among Taiwanese CPV-2 isolates (94.5 %), and our results revealed that this variant is not only present among Taiwanese CPV-2a strains but also CPV-2b and -2c strains. In addition, this study reports for the first time the amino acid substitution of Phe267Tyr in Taiwan. Surprisingly, all of the Tyr267 variants among Taiwanese CPV-2a, −2b, and −2c strains contain the amino acid substitution of Tyr324Ile. Our review of sequence analysis in the literature indicated that this phenomenon is also found in Uruguay [51]. The functions of CPV-2 residues 267 and 324 are still unknown and remain to be elucidated.

The substitution of Gln370Arg is unique to the Taiwanese CPV-2c strains, and this mutation is also observed in Chinese panda parvovirus [52] and Chinese CPV-2c strains [26, 27]. Residue 359 and 375 constitute a flexible surface loop of the capsid protein that is adjacent to a double Ca^2+^-binding site; this region is essential for virus infectivity, and changes are correlated with the ability of the virus to cause erythrocyte hemagglutination [53]. Therefore, it remains to be investigated whether Gln370Arg substitution causes antigenic alterations.

Mutation of residue 420 had been reported in Brazilian reference CPV-2c strains (Phe420Leu) [54]. In the present study, we detected 10 CPV-2c strains with a unique change at the same position, yet Phe420Ser was unique in these 10 Taiwanese CPV-2c strains. Therefore, further studies focusing on potential variants of the CPV-2c strains should be conducted to elucidate the relationship between Phe420Ser substitution and viral pathogenicity.

Although several studies have demonstrated the efficacy of the current CPV-2 vaccine against CPV-2c infection [55, 56], some evidence suggests that dogs with the complete vaccination program still suffer from CPV-2c [6]. In the present study, four of 22 CPV-2c-diseased dogs died despite vaccination (C104-030, C104-031, C104-042, C104-216) (Additional file 1). Among those that died, three were under 6 months of age. Surprisingly, despite having undergone the complete vaccination program, one adult dog (strain no. C104-216) was infected by this novel CPV-2c variant. Therefore, co-infection with other diseases needs to examined, and the efficacy of the current vaccine against this novel CPV-2c variant remains to be evaluated, especially in regard to the amino acid substitutions observed in this novel CPV-2c variant compared to the CPV-2c prototype.

## Conclusions

This is the first report to identify a novel CPV-2c variant in Taiwan. The novel CPV-2c variant was found to be distributed throughout Taiwan, revealing that this novel CPV-2c variant is currently circulating on the island. Phylogenetic analysis demonstrated that the recent CPV-2c isolate from Taiwan shares a common evolutionary origin with Chinese strains of CPV-2c, as classified into novel Asian CPV-2c isolates (Phe267Tyr, Tyr324Ile, Gln370Arg). Continuous and intensive surveillance of this novel CPV-2c is needed, especially in previously disease-free countries.

## Additional file

Additional file 1: The genotypes of 88 canine parvovirus type 2 isolates collected from Taiwanese dogs. (DOCX 74 kb)

## Abbreviations

CPV-2: Canine parvovirus type 2
CPV-2c: Canine parvovirus type 2c
PCR: Polymerase chain reaction
