# Supplementary material for: Identification of a novel canine parvovirus type 2c in Taiwan
Source: Virol J. 2016 Sep 23;13:160. doi: 10.1186/s12985-016-0620-5 (PMC5035481; doi:10.1186/s12985-016-0620-5)
Supplement: Additional file 1: — The genotypes of 88 canine parvovirus type 2 isolates collected from Taiwanese dogs. (DOCX 74 kb) [file 12985_2016_620_MOESM1_ESM.docx]

Additional file 1 The genotypes of 88 canine parvovirus type 2 isolates collected from Taiwanese dogs

| Collection date | Strain | Genotype | Region | Age | Sex | Vaccination | Death | Accession number |
| --- | --- | --- | --- | --- | --- | --- | --- | --- |
| 2014.03.11 | C103-012 | 2b | South | N/A^a^ | N/A | N/A | N/A | KX396348 |
| 2014.04.05 | C103-015 | 2a | South | N/A | N/A | N/A | N/A | KX396349 |
| 2014.05.02 | C103-021 | 2b | South | N/A | N/A | N/A | N/A | KX396350 |
| 2014.05.27 | C103-028 | 2b | South | N/A | N/A | N/A | N/A | KX396351 |
| 2014.05.27 | C103-029 | 2b | South | N/A | N/A | N/A | N/A | KX396352 |
| 2014.07.15 | C103-035 | 2a | South | N/A | N/A | N/A | N/A | KX396353 |
| 2014.12.04 | C103-107 | 2a | South | N/A | N/A | N/A | N/A | KX396354 |
| 2015.01.15 | C104-014 | 2c | South | 3 M | M | Yes | No | KX396355 |
| 2015.03.05 | C104-019 | 2a | South | 2 M | N/A | N/A | Yes | KX396356 |
| 2015.04.13 | C104-030 | 2c | Central | N/A | F | Yes | Yes | KX396357 |
| 2015.04.13 | C104-031 | 2c | Central | N/A | N/A | Yes | Yes | KX396358 |
| 2015.04.21 | C104-037 | 2a | South | 3 M | F | Yes | No | KX396359 |
| 2015.05.01 | C104-042 | 2c | Central | 3 M | F | Yes | Yes | KX396360 |
| 2015.05.12 | C104-046 | 2b | Central | 3 M | M | No | No | KX396361 |
| 2015.05.15 | C104-049 | 2c | South | 1 Y | M | No | No | KX396362 |
| 2015.05.18 | C104-051 | 2c | South | N/A | N/A | N/A | N/A | KX396363 |
| 2015.05.18 | C104-052 | 2c | South | N/A | N/A | N/A | N/A | KX396364 |
| 2015.05.21 | C104-053 | 2a | South | 5 M | M | No | No | KX396365 |
| 2015.05.27 | C104-055 | 2c | North | 4 M | M | No | N/A | KX396366 |
| 2015.05.27 | C104-057 | 2c | North | 7 M | M | Yes | No | KX396367 |
| 2015.06.08 | C104-070 | 2b | North | 1 M | F | No | N/A | KX396368 |
| 2015.06.08 | C104-071 | 2b | North | 2.5 M | M | Yes | N/A | KX396369 |
| 2015.06.08 | C104-072 | 2b | North | 2 M | M | Yes | N/A | KX396370 |
| 2015.06.08 | C104-073 | 2b | North | 1.5 M | F | No | N/A | KX396371 |
| 2015.07.15 | C104-107 | 2b | North | 2 M | M | Yes | N/A | KX396372 |
| 2015.07.15 | C104-108 | 2b | North | 1.5 M | F | Yes | N/A | KX396373 |
| 2015.07.15 | C104-109 | 2b | North | 1.5 M | F | No | N/A | KX396374 |
| 2015.08.25 | C104-143 | 2a | East | 3 M | M | Yes | Yes | KX396375 |
| 2015.08.26 | C104-144 | 2a | East | 3 Y | F | Yes | No | KX396376 |
| 2015.08.31 | C104-154 | 2a | Central | 2.5 M | F | No | No | KX396377 |
| 2015.08.31 | C104-155 | 2a | Central | 2.5 M | F | No | No | KX396378 |
| 2015.09.05 | C104-165 | 2c | North | 1 M | F | No | No | KX396379 |
| 2015.09.05 | C104-166 | 2c | North | 1 M | M | No | Yes | KX396380 |
| 2015.09.05 | C104-167 | 2c | North | 1 M | F | No | Yes | KX396381 |
| 2015.09.14 | C104-171 | 2b | East | 5 M | F | No | No | KX396382 |
| 2015.10.11 | C104-211 | 2b | North | 9 M | M | No | No | KX396383 |
| 2015.10.14 | C104-216 | 2c | South | 11 M | F | Yes | Yes | KX396384 |
| 2015.10.17 | C104-217 | 2a | East | 5 M | F | No | Yes | KX396385 |
| 2015.10.25 | C104-225 | 2b | Central | 3 M | N/A | No | N/A | KX396386 |
| 2015.10.25 | C104-226 | 2b | Central | 3 M | N/A | No | N/A | KX396387 |
| 2015.11.09 | C104-238 | 2a | East | 3 M | F | No | No | KX396388 |
| 2015.11.18 | C104-248 | 2c | South | 6 M | M | No | No | KX396389 |
| 2015.11.23 | C104-250 | 2a | East | 3 M | F | No | No | KX396390 |
| 2015.12.09 | C104-263 | 2c | Central | 8 M | M | Yes | N/A | KX396391 |
| 2015.12.09 | C104-264 | 2c | Central | 6 M | M | No | No | KX396392 |
| 2015.12.09 | C104-265 | 2c | Central | 3 M | M | No | N/A | KX396393 |
| 2015.12.09 | C104-266 | 2c | Central | 17 M | F | Yes | No | KX396394 |
| 2015.12.09 | C104-267 | 2c | Central | 3 M | M | No | No | KX396395 |
| 2015.12.11 | C104-269 | 2c | East | 2 M | N/A | No | No | KX396396 |
| 2015.12.11 | C104-270 | 2c | East | 6 M | N/A | No | No | KX396397 |
| 2015.12.11 | C104-271 | 2c | Central | 3 M | N/A | No | Yes | KX396398 |
| 2015.12.11 | C104-272 | 2c | South | 6 M | F | No | No | KX396399 |
| 2015.12.14 | C104-273 | 2a | East | 5 M | F | No | Yes | KX396400 |
| 2015.12.14 | C104-275 | 2c | South | 4 M | M | No | Yes | KX396401 |
| 2015.12.18 | C104-279 | 2c | South | N/A | N/A | N/A | N/A | KX396402 |
| 2015.12.23 | C104-283 | 2c | Central | 4 M | F | No | Yes | KX396403 |
| 2016.01.04 | C105-002 | 2a | East | 1 Y | F | No | No | KX396404 |
| 2016.01.12 | C105-010 | 2b | Central | 4 M | F | Yes | Yes | KX396405 |
| 2016.01.12 | C105-011 | 2b | Central | 4 M | M | Yes | No | KX396406 |
| 2016.01.12 | C105-012 | 2b | Central | 4 M | F | Yes | No | KX396407 |
| 2016.01.12 | C105-013 | 2c | East | 2 M | N/A | No | Yes | KX396408 |
| 2016.01.13 | C105-014 | 2b | Central | 4 M | M | Yes | No | KX396409 |
| 2016.01.13 | C105-015 | 2c | North | 6 M | M | Yes | N/A | KX396410 |
| 2016.01.13 | C105-016 | 2c | North | 3 M | M | No | Yes | KX396411 |
| 2016.01.13 | C105-017 | 2c | North | 4 M | F | No | Yes | KX396412 |
| 2016.01.13 | C105-018 | 2c | South | 3 M | F | No | No | KX396413 |
| 2016.01.18 | C105-019 | 2c | East | 4 M | F | No | No | KX396414 |
| 2016.01.29 | C105-022 | 2a | East | 7 M | F | No | No | KX396415 |
| 2016.02.17 | C105-027 | 2c | Central | 2 M | M | No | Yes | KX396416 |
| 2016.02.20 | C105-029 | 2b | North | 2 M | F | No | No | KX396417 |
| 2016.02.21 | C105-030 | 2c | Central | N/A | M | No | Yes | KX396418 |
| 2016.02.25 | C105-034 | 2a | South | 2 M | F | No | Yes | KX396419 |
| 2016.02.26 | C105-035 | 2b | North | 2 M | M | No | No | KX396420 |
| 2016.03.08 | C105-038 | 2b | Central | 4 M | M | Yes | N/A | KX396421 |
| 2016.03.08 | C105-039 | 2c | North | 4 M | F | No | No | KX396422 |
| 2016.03.13 | C105-042 | 2c | Central | 2 M | F | No | Yes | KX396423 |
| 2016.03.15 | C105-044 | 2c | South | 4 M | M | No | Yes | KX396424 |
| 2016.03.15 | C105-045 | 2c | South | 3 M | F | No | No | KX396425 |
| 2016.03.16 | C105-046 | 2c | Central | 2 M | N/A | No | Yes | KX396426 |
| 5016.03.22 | C105-050 | 2c | South | 4 M | M | No | No | KX396427 |
| 2016.03.29 | C105-056 | 2c | Central | 3 M | M | No | Yes | KX396428 |
| 2016.03.29 | C105-057 | 2c | Central | 3 M | M | No | Yes | KX396429 |
| 2016.03.29 | C105-058 | 2c | Central | 3 M | F | No | Yes | KX396430 |
| 2016.03.29 | C105-059 | 2c | Central | 3 M | M | No | Yes | KX396431 |
| 2016.04.13 | C105-067 | 2c | Central | 3 M | F | No | No | KX396432 |
| 2016.04.27 | C105-072 | 2c | East | 3 M | F | No | No | KX396433 |
| 2016.04.29 | C105-074 | 2c | East | 3 M | F | No | No | KX396434 |
| 2016.04.29 | C105-075 | 2c | North | 6 M | M | No | Yes | KX396435 |

^a^N/A: not available.
